# Does sleep promote adaptation to acute stress: An experimental study

**DOI:** 10.1016/j.ynstr.2024.100613

**Published:** 2024-02-04

**Authors:** Emil Hein, Risto Halonen, Thomas Wolbers, Tommi Makkonen, Markus Kyllönen, Liisa Kuula, Ilmari Kurki, Philipp Stepnicka, Anu-Katriina Pesonen

**Affiliations:** a SleepWell Research Program, Faculty of Medicine, University of Helsinki, Helsinki, Finland; b Deutsches Zentrum für Neurodegenerative Erkrankungen (DZNE), Magdeburg, Germany; c Neomento GmbH, Berlin, Germany; d Department of Psychology and Logopedics, Faculty of Medicine, University of Helsinki, Helsinki, Finland

**Keywords:** Sleep, Stress, Polysomnography, Experimental study, Virtual reality

## Abstract

**Objectives:**

Evidence of the impact of chronic stress on sleep is abundant, yet experimental sleep studies with a focus on acute stress are scarce and the results are mixed. Our study aimed to fill this gap by experimentally investigating the effects of pre-sleep social stress on sleep dynamics during the subsequent night, as measured with polysomnography (PSG).

**Methods:**

Thirty-four healthy individuals (65% females, M_age_ = 25.76 years SD = 3.35) underwent a stress-inducing (SC) or neutral control condition (CC) in virtual reality (VR). We used overnight EEG measurements to analyze the basic sleep parameters and power spectral density (PSD) across the sleep cycles, and measured heart rate and its variability (HRV), skin electrodermal activity (EDA), and salivary cortisol to capture physiological arousal during the VR task and the pre-sleep period.

**Results:**

Following acute stress (SC), the amount of slow-wave sleep (SWS) was higher and N2 sleep lower relative to CC, specifically in the first sleep cycle. In SC, PSD was elevated in the beta-low (16–24 Hz) and beta-high (25–35 Hz) frequency ranges during both stages N2 and SWS over the entire night.

**Conclusions:**

Sleep promoted adaptation to acute social stress by a longer duration of SWS in the subsequent sleep period, especially in early sleep. A similar homeostatic effect towards restorative sleep is well-evidenced in animal model stress studies but has not been previously reported in experimental human studies. Whether the high-frequency PSD activity during stages N2 and SWS also serves in the resolution of transient stress, remains open.

## Highlights

• Acute social stress induces slow-wave rebound sleep in humans.
• The slow-wave sleep rebound occurs simultaneously with high frequency EEG activity.
• Both the sleep rebound and cortical arousal may function adaptively to acute stress.
• Our results converge with experimental stress studies on animal models.

## 1 Introduction

Experiences of acute stress are ubiquitous and unavoidable throughout life. Acute stress induces heightened states of vigilance, usually involving somatic (i.e. hormonal, cardiovascular) changes and cortical hyperarousal, (Campbell and Ehlert, 2012) which, evolutionarily, support adaption to environmental challenges (Badyaev, 2005). Regarding sleep however, the stress response may result in maladaptive outcomes as pre-sleep hyperarousal is suggested to shorten sleep and compromise sleep quality (Riemann et al., 2010). As pre-sleep hyperarousal is a central concept in primary insomnia (Riemann et al., 2010; Perlis et al., 1997) and interacts with long-term comorbidities, such as mood disorders (Goldstein and Walker, 2014; Riemann et al., 2020) and burnout, (Ekstedt et al., 2006) it constitutes a global public health concern (Luyster et al., 2012). Yet, the sleep research focus in humans has mainly centered on the pathological development of chronic stress conditions, and consequently, the experimental evidence on acute modes of stress in relation to sleep in healthy individuals is scarce (Pesonen et al., 2021; Kim and Dimsdale, 2007).

Current research evidence on healthy adults shows that experiencing acute stress may alter subsequent sleep periods, but the consequences are not yet consolidated due to considerable variability in the study designs, results, and evaluated outcomes (Kim and Dimsdale, 2007). In experimental studies, acute stress is typically induced through psychosocial stress tasks designed to elicit negative self-conscious affect in participants (i.e. nervousness, shame), (Dickerson et al., 2004) as in the commonly used Trier Social Stress Test (TSST) (Campbell and Ehlert, 2012; Kirschbaum et al., 1993). Other methods to induce social stress include listening to one's own out-of-tune karaoke singing, (Wassing et al., 2019) arithmetic tasks with negative feedback, (Dammen et al., 2022) or engaging in a virtual dance competition (van Dammen et al., 2020). Common between these studies, they all include a social-evaluative threat and the impression of uncontrollability to generate a stress experience, argued to be the key characteristics of social stress (Dickerson et al., 2004; Dickerson and Kemeny, 2004). Social stress experiences have been demonstrated to be validly simulated in virtual reality (VR) environments as well, (Kothgassner et al., 2016; Diemer et al., 2015) including virtual adaptations of the TSST (Zimmer et al., 2019).

Previous experimental studies utilizing pre-sleep stress induction report varying outcomes. The most common observation shows a prolonged sleep onset latency (SOL) (Zoccola et al., 2009; Wuyts et al., 2012; Ackermann et al., 2019) or a decrease in sleep efficiency (SE) (Beck et al., 2022; Vandekerckhove et al., 2011). Regarding the proportion of sleep stages, the results are mixed. While most studies have not reported changes in the organization of sleep stages, (Kim and Dimsdale, 2007; Wuyts et al., 2012; Ackermann et al., 2019; Kim et al., 2020) one study noted reduced rapid eye movement sleep (REMS) (Vandekerckhove et al., 2011) and another reduced SWS and N2 (Beck et al., 2022) following pre-sleep stress. Except for one study, (Kim et al., 2020) all pre-sleep stress studies involving analysis of power spectral density (PSD) of the EEG signal have reported significant changes in delta and/or beta power. Subsequent to the stress induction, either only delta power decreased (Ackermann et al., 2019) or the decrease coincided with an increase in beta power (Wuyts et al., 2012; Beck et al., 2022). Increased beta power during sleep has been associated with poorer sleep quality, (Krystal and Edinger, 2008) hyperarousal in insomnia, (Riemann et al., 2010; Maes et al., 2014) and psychological stress burden (Hall et al., 2007) in a plethora of non-experimental studies. Noteworthy in the abovementioned experimental studies with PSD, two are based on a napping design (Ackermann et al., 2019; Beck et al., 2022) and two on an overnight sleep assessment (Wuyts et al., 2012; Kim et al., 2020). The latter allows better tracking of the temporal dynamics of sleep experienced in everyday life, as overnight measures align better with the circadian and homeostatic drivers of sleep.

The current study aims to contribute further to the understanding of the effects of acute stress on sleep over the entire night. To this end, we applied a real-life simulating public speaking stress scenario in VR to induce acute social stress during the pre-sleep period and to investigate its impact on sleep (stress condition, SC). We compared the effect with a control condition (CC), comprising a passive listening task in VR. This study utilized PSD analysis to evaluate the temporal sleep EEG dynamics over an entire night, reflecting neural activity during sleep, and giving further insight into sleep quality beyond the basic sleep parameters. We used naturally occurring sleep cycles as the repeated measures factor in the investigation of EEG dynamics, analyzed in six frequency ranges for central and frontal topologies during non-REM sleep (NREMS) and REMS. This approach has not been previously reported, and it provides a holistic overview of the sleep dynamics after an experience of acute stress. Our main hypothesis states that there is elevated beta activity and/or decreased delta/beta ratio during sleep subsequent to stress, following the hyperarousal model of insomnia, (Riemann et al., 2010) and also evidenced in a napping study on healthy participants (Beck et al., 2022). However, in overnight studies on acute social stress, supporting evidence is currently scarce (Kim et al., 2020). In addition, based on animal model evidence, a restorative sleep rebound effect would be anticipated, (Fujii et al., 2019; Meerlo and Turek, 2001; Wells et al., 2017; Yu et al., 2022) although this has not yet been evidenced in studies in humans.

## 2 Participants and methods

### 2.1 Sample and study design

**Fig. 1:**
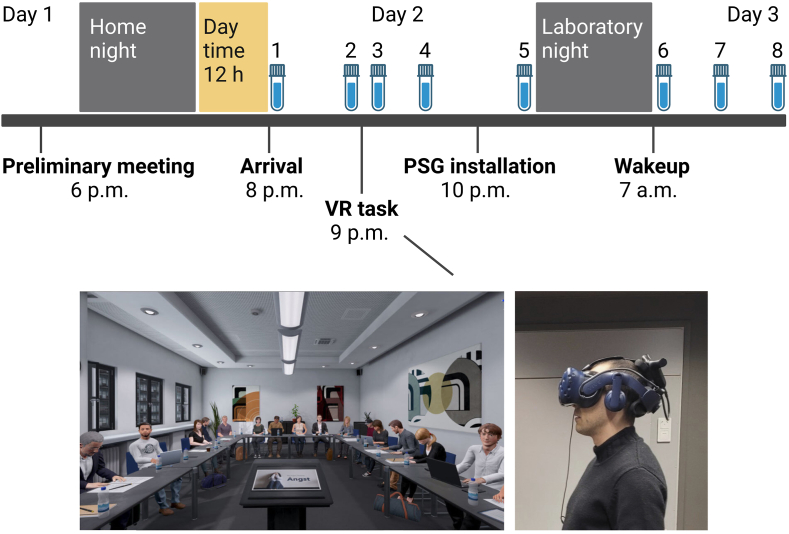
Study design. We introduced the participants to the protocol during the preliminary meeting on *day 1,* assigning their experimental conditions and installing the beat-to-beat and electrodermal activity monitoring devices. Participants spent the subsequent night at home. On *day 2*, participants spent the daytime freely, arrived at the sleep laboratory at 8 p.m., and completed the VR experiment prior to overnight sleep polysomnography (PSG) measurement in the laboratory. Sleep opportunity was set between 11 p.m. and 7 a.m. One hour after awakening, participants evaluated their sleep quality, and all devices were removed. Cortisol samples and their numbers are indicated by the vial illustrations. Based on averaged times, sample 1 was taken at 08:10 pm. upon arrival at the laboratory. Sample 2 was taken at 08:50 p.m. as a baseline before the VR task. Sample 3 was taken immediately after task completion, and sample 4 was timed exactly 20 min after sample 3 to capture the post-task cortisol peak. Pre-sleep cortisol was assessed with sample 5, taken on average at 11:50 p.m. right before lights off. The mean interval between samples 4 and 5 was 2 h 14 min (SD = 14 min). To assess the cortisol awakening response, samples 6, 7, and 8 were taken on *day 3* immediately after waking up at 7 a.m., and 30 and 60 min thereafter.

The sample consisted of thirty-four Finnish-speaking young adult volunteers (65% females, M_age_ = 25.76 years, standard deviation SD = 3.35) recruited via university student mailing lists within the Helsinki capital area (Table 1). Exclusion criteria for participation were sleeping disorders, use of medication affecting sleep, and transcontinental travel within the past three weeks. The participants were instructed to abstain from alcohol throughout the study. Caffeine was restricted on the day of overnight measurement from 4 p.m. onwards and nicotine from 8 p.m. onwards. We also instructed the participants to keep a regular sleeping schedule and a sleep diary on the bed and wake up times (15 min (min) accuracy) four nights before the measurement night. Napping was discouraged throughout the study. Participants signed an informed consent form before participation and received monetary compensation (€100) for completing the study. Helsinki University Hospital Ethics Committee approved our protocol, and we conducted all components of the study in adherence to the Declaration of Helsinki and its later amendments. We collected the data between May 2021 and January 2022. Fig. 1 illustrates the 2-day procedure and study design.

**Table 1:** Sample characteristics, questionnaires, and self-reports by the condition.

	SC		CC		p^a^
	Mean	SD	Mean	SD	
Sample characteristics					
n (F)	20 (12)	na	14 (10)	na	.493
Age	26.66	3.16	24.47	3.40	.063
BMI	22.69	2.30	23.57	2.52	.298
Questionnaires					
PSQI	8.45	2.63	7.57	1.99	.416
BDI-II	7.50	6.86	5.36	5.05	.416
SPIN	7.85	6.17	11.36	9.07	.306
GAD-7	3.70	3.10	2.36	2.41	.148
Self-ratings					
VR immersion score	4.37	1.2	4.71	0.9	.304
VR stressfulness score	3.68	1.2	1.14	0.4	**<.001**
Sleep quality	3	0.6	2.9	0.5	.592

n = number, F = female, SC = stress condition, CC = control condition, BMI = body mass index, PSQI = Pittsburgh Sleep Quality Index, BDI-II = Beck Depression Inventory II, SPIN = Social Phobia Inventory, GAD-7 = Generalized Anxiety Disorder 7 Assessment, VR = virtual reality, SD = standard deviation.

a Chi-square test for sex distribution, unadjusted independent *t*-test for age and BMI, independent samples Mann-Whitney *U* test for questionnaires and self-ratings.

#### 2.1.1 Experimental VR task

Each participant completed an experimental VR task, a scenario in a virtual seminar room (Fig. 1), using the neomento VR software (neomento GmbH, https://neomento.de), which has been developed as an immersive exposure therapy tool for anxiety disorders (Streck et al., 2019). The stress condition scenario comprised an unprepared oral presentation (M_duration_ = 7.92 min, SD = 1.77 min) in front of a virtual audience guided by four time-gated slides on the speaker's podium. Each slide presented a set of personal questions of increasing emotional salience about the participant's life and opinions (e*.g. I am different from other because …, I was ashamed when …*), while an interval timer sound every 2 min indicated the answering time limit to change to the next slide. If the participant had finished their answers before the preset time for each slide was over, they were encouraged to continue talking to the audience. Before the SC scenario, we disclosed that their performance would be evaluated and compared with that of other participants. To further elevate the social-evaluative threat, the virtual audience was programmed to appear focused on the participant during the first half of the presentation but started to show signs of disinterest (i.e. looking out of the window, playing with a laptop) during the second half. To minimize uncanny valley effects, the virtual humans displayed randomized blinking and occasionally switched to a different action based on a probabilistic state machine (Streck and Wolbers, 2018). Finally, in CC participants passively listened to a neutral presentation entitled “Mineral Types in Finnish Nature” (fixed duration 6 min), displayed on a screen in the same virtual seminar room as in SC, but without the virtual audience.

Participants sat during the baseline and postline periods, but both VR conditions were completed while standing up. All participants completed a guided VR device tutorial (M_duration_ = 4.85 min, SD = 1.05 min) before either task. The VR equipment that we used included an HTC Vive Pro VR Headset and HTC Vive Pro Controllers (HTC Corporation, https://www.vive.com) with the VR headset tracking performed using two SteamVR Base Station 2.00 units powered by SteamVR software (Valve Corporation, https://store.steampowered.com) on a Windows PC (Graphics: MSI GeForce RTX, 2080).

### 2.2 Measures

#### 2.2.1 Questionnaires and self-ratings

Sample trait characteristics were assessed with BDI-II (Beck et al., 1988), GAD-7 (Spitzer et al., 2006), and SPIN (Connor et al., 2000) questionnaires, and sleep disturbances with PSQI (Buysse et al., 1989). Approximately 1.5 h after the VR task, participants evaluated their experience on a scale of 1–7 for the believability of the VR environment (very unconvincing to real) and stressfulness of the experience (none to extremely stressful). The next morning, all participants evaluated their sleep quality on a scale of 1–4 (poor to very good), and again, the SC group estimated their emotions felt during the public speaking VR task (yes/no for being evaluated, social pressure, embarrassment, being judged). All participants estimated their sleep duration, bedtimes and waking up times during four days before the laboratory night.

#### 2.2.2 Physiological measures

**Cortisol.** We collected eight salivary cortisol samples (Fig. 1) to assess reactivity of the hypothalamic-pituitary-adrenal axis to the VR task and to capture the cortisol awakening response. Salivette cotton swabs (Sarsted, https://www.sarstedt.com) were used, and we instructed participants to hold the cotton swab for 2 min in their mouth and to avoid drinking or eating for at least 30 min before each sample. Samples were stored at −20 °C and analyzed at the University of Trier cortisol laboratory. Cortisol concentration of each sample was measured twice, and their average nmol/l value was used in analyses. Data from one participant (SC) were rejected due to outlier values. Using raw values, we calculated task peak reactivity (task peak - baseline), cortisol awakening response (CAR; morning peak - first morning sample) and time-weighted area under the curves (AUC_I_) for both evening and morning samples (Pruessner et al., 2003). For linear modelling, we transformed raw cortisol values with natural logarithm to attain normality.

**HR and HRV.** Heart rate (HR) and its variability (HRV) were assessed with Firstbeat Bodyguard 2 (Firstbeat Technologies Oy, https://www.firstbeat.com) beat-to-beat heart rate monitoring device (Parak and Korhonen, 2015) with a sampling rate of 1000 Hz. The device was attached with adhesive electrodes (Covidien Kendall H92SG ECG, https://www.medtronic.com), one placed under the right collarbone and the second under the left costal arch. We performed Fast Fourier Transformations (FFT) on data from the VR task period (baseline: 20 min before task; peak: 20 min after task onset; postline: 20 min after peak) and on data from the pre-sleep period (30 min before lights off + SOL) in Kubios HRV Scientific 4.0.2. (Kubios Oy, https://www.kubios.com) using its default FFT settings. The signal power spectrum was split into high-frequency (HF, 0.15–0.4 Hz), low-frequency (LF, 0.04–0.15 Hz) and very-low-frequency (VLF, 0.00–0.04 Hz) components, reported in relative powers. Artifacts were rejected with Kubios’ built-in noise detection (medium filtering) and beat correction algorithms (Lipponen and Tarvainen, 2019). Data from two participants (both SC) were corrupt due to electrode contact error.

**EDA.** Electrodermal activity (EDA) was measured during the VR task with Moodmetric EDA rings (Jussila et al., 2018) (re-branded Nuanic ring, Nuanic Oy, https://nuanic.com) with sampling rates of 3 Hz. The minute-specific baseline was measured during the VR device tutorial (see 2.1.1. Experimental VR task), peak during the VR task, and postline 5 min after the task. Participants wore the ring on the non-dominant hand. Due to technical issues, several EDA measurements were rejected (7 SC, 4 CC).

#### 2.2.3 Polysomnography and sleep cycles

We measured sleep with an EEG system and software by Brain Products (Brain Vision LLC, https://brainvision.com): Easycap Standard 128Ch cap, two actiCAP snap 32-channel active electrode bundles, actiCHamp Plus amplifier, ground electrode located at Fpz. Electro-oculograms (EOGs) and electromyograms (EMGs) were measured with electrodes from one of the EEG bundles: EMG attached to the right upper back trapezius muscle, EOG1 attached to the right cheek, and EOG2 kept in the cap above the left eye. Sampling rate was 500 Hz. The online reference electrode was Cz but data was re-referenced to FCz before sleep scoring and analysis. We scored the polysomnography data into N1, N2, SWS (N3), REMS, and wake stages, according to the AASM 2.6 (American Academy of Sleep Medicine, https://aasm.org). Epochs containing arousals or signs of wakefulness longer than 15 s were marked to be excluded from the PSD analysis. We defined sleep cycles based on rules adapted from Feinberg and Floyd (1979) such that NREMS and REMS cycles were combined into general sleep cycles. In some cases, we corrected the durations of the sleep cycles (33/136 adjusted, 24.3%) as follows: Exceptionally long sleep cycles (>140 min) were split in two, such that any SWS or REMS episodes were not interrupted. Short sleep cycles (<70 min) were extended past the REMS episode to a minimum of 70 min, on the condition that subsequent SWS episode was not included. Each correction was based on the respective hypnogram, such that SWS and REMS episodes defining a sleep cycle were preserved. In addition, at least 15 min of interim stage N2 or SWS was required to separate fragmented REMS into separate REMS episodes. Wake periods longer than 15 min split the sleep cycles. The final sleep cycle before waking up was merged with the previous cycle if its duration was less than 15 min. These corrections led to an average of 19 and 20 min (SDs = 15 min) changes in absolute cycle durations in SC and CC, respectively (independent *t*-test between the conditions p = .884).

#### 2.2.4 EEG power spectral density analysis

We performed FFT on the raw EEG data, completed with Matlab R2021b (MathWorks Inc., https://www.mathworks.com) using the EEGLAB function spectopo (Delorme and Makeig, 2004). Pre-processed signal length was 30 s (s) with pre-processing filters for high-pass at 0.5 Hz and low-pass at 40 Hz. FFT window size was set at 2000 and window overlap at 50%. Separately for each participant, sleep stage, electrode, and sleep cycle, we exported mean PSD values of each 30 s epoch. We chose frequency bins for six ranges: delta (0.5–3 Hz), theta (4–7 Hz), alpha (8–12.5 Hz), sigma (9–16 Hz), beta-low (16–24 Hz), and beta-high (25–35 Hz). Artifact epochs were systematically rejected in two parts. First, individual epochs (from one or more electrodes) were rejected based on either absolute power value, SD, skewness or kurtosis by a 3 SD criterion, calculated per sleep stage individually for each participant (rejection rate 3.25%). Individual outlier PSD values left in the dataset were rejected using a stricter 2 SD criterion, calculated per frequency range (rejection rate 14.16%). Data from three participants (2 SC, 1 CC) were fully rejected from PSD analysis due to reference electrode contact failure. The final dataset was normalized using Min-Max normalization. For the current study questions, electrodes Fz, F3, F4, Cz, C3, and C4 were chosen and we averaged adjacent electrodes into frontal and central regions separately for each frequency band, in case data points from at least two electrodes were available (86% with three electrodes, 3% with two electrodes; 11% all electrodes missing). Finally, we calculated delta/beta ratios (beta = beta-low + beta-high)/2), which attenuate the statistical impact of individual baseline PSD differences. Topographical scalp maps were visualized using the topoplot function of YASA 0.6.3 (https://raphaelvallat.com/yasa) in Python 3.7 (https://www.python.org).

### 2.3 Statistical analyses

We performed all analyses in IBM SPSS Statistics 29.0 (IBM Corporation, https://www.ibm.com). Alpha levels (two-tailed) were set at p < .05 for significance and confidence interval at 95% for all models. Due to their influence on physiology and sleep characteristics, (Ohayon et al., 2004; Baker and Driver, 2007) all tests and models were adjusted, unless inapplicable or specified, for age and sex (assigned at birth, sample distribution binary) by using them as covariates in the models. There was no collinearity between sex, age and experimental condition (all VIF < 1.25). We tested differences between the conditions, SC and CC, in the following analyses. First, we analyzed sample characteristics: BMI and age with unadjusted independent samples *t*-test, sex distribution with chi-square test, and questionnaires and self-ratings with independent samples Mann-Whitney *U* test. Second, we used linear mixed models (REML method, random ID intercept, time as the repeated observation with first-order autoregressive covariance structure) to study how the condition affected heart rate and its variability, EDA and log transformed cortisol values during the evening (main effect and ‘time x condition’ interaction). We also studied the main effect and ‘time x condition’ interaction for morning cortisol levels. In addition, we used multivariate ANOVA to study cortisol AUC_I_, task peak reactivity, and CAR. Finally, differences in basic sleep parameters between SC and CC were studied with multivariate ANOVA and differences in PSD during sleep studied with linear mixed models (main effect and ‘time x condition’ interaction, REML method, random ID intercept, sleep cycle (4 total) as the repeated observation with diagonal covariance structure). We ensured mixed model fits with Akaike's information criterion. Sensitivity analysis on G*Power (Faul et al., 2007) determined our total sample size of 34 to reliably detect large effect sizes in all selected statistical models at 80% power.

## 3 Results

### 3.1 Initial analyses

#### 3.1.1 Sample characteristics

The samples of the experimental conditions did not differ in age, sex distribution or body mass index (all p > .06, Table 1). There were no condition differences in any of the questionnaire or self-report scores, except for the higher VR task stressfulness self-ratings in SC (U = 3.000, Z = −4.907, p < .001). The 20 participants in SC were given a yes/no questionnaire in the following morning and a positive answer was reported in the following categories: being evaluated 16 (80%), experiencing social pressure 12 (60%), feeling embarrassed 9 (45%), and being judged 8 (40%). The sleep durations during the four nights before the experiment were not significantly different between the conditions (Supplement Table 2).

#### 3.1.2 Cortisol stress response

**Fig. 2:**
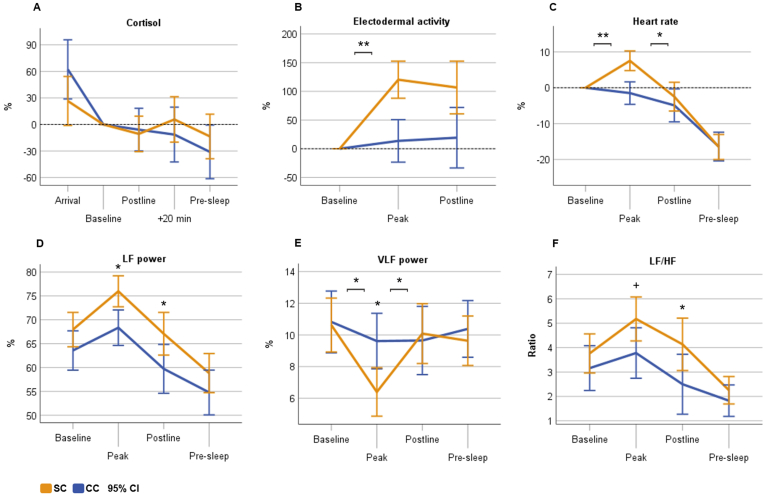
Physiological measures during the laboratory evening. Cortisol (A), heart rate (B), and electrodermal activity (C) expressed in relative proportion to the baseline segment, and all figures are adjusted for sex and age. LF = low-frequency, VLF = very-low-frequency, HF = high-frequency, SC = stress condition, CC = control condition, bars refer to 95% confidence interval (CI). **p < .001, *p < .05, +p < .07 linear mixed model condition pairwise comparison and ‘time x condition’ interaction contrasts.

The SC had less steep decrease of cortisol from arrival to the laboratory to the pre-sleep sample as measured from the AUC_I_ (F(1,29) = 4.641, p = .040, η^2^ = 0.138), but the condition main effect, task peak reactivity, and the interaction ‘time x condition’ during the evening were non-significant (all p > .10) (Fig. 2A). During the next morning, both CAR and AUC_I_ as well as the condition main effect and the ‘time x condition’ interaction were all non-significant between the conditions (all p > .45).

#### 3.1.3 HR, HRV and EDA stress response

Table 2 displays the other physiological measures across the study timeline, and Fig. 2 (B–F) illustrates the reactivity measured from different time points. We observed significant main effects for SC in the HRV measures LF (F(1,27.999) = 6.903, p = .014) and LF/HF (F(1,27.442) = 4.380, p = .046). Regarding the post-hoc tests, the condition differences were specifically observed during task peak in LF (M_diff_ = 7.102, F(1,70.715) = 6.066, p = .016) and VLF (M_diff_ = −3.677, F(1,71.104) = 8.761, p = .004). During postline, the conditions differed significantly in LF (M_diff_ = 7.000, F(1,70.715) = 5.893, p = .018), HF (M_diff_ = −7.452, F(1,63.436) = 5.507, p = .022), and in LF/HF (M_diff_ = 1.586, F(1,79.101) = 6.170, p = .015). Regarding the ‘time x condition’ interactions, there were significant interactions for HR (p = .004), EDA (p < .001) and VLF (p = .009) measures. Investigation of the contrasts between the time points showed that there was a significant change from baseline to task peak in HR (B = 7.020, t(83.092) = 3.577, p < .001), VLF (B = −3.715, t(73.389) = −2.846, p = .006) and in EDA (B = 3.058, t(43.636) = 4.062, p < .001) for SC compared with CC. There was a significant post-stress recovery from task peak to postline in HR (B = −4.757, t(83.092) = −2.424, p = .018) and VLF (B = 4.154, t(73.389) = 3.182, p = .002) but not in EDA (p = .247). Regarding the values in the pre-sleep period, the conditions did not differ significantly (all p > .08, Table 2).

**Table 2:** Linear mixed models between the conditions for the physiological parameters across the entire evening (from the baseline to pre-sleep).

	Main effect for SC		Time x condition		Condition difference in time points^a^^,^^b^ A/B/C/D	Time x condition contrasts^a^ A vs. B/B vs. C/C vs. D
	B [95% CI]	p	F (df)	p	p	p
Mean HR (bpm)	−2.60 [−9.89, 4.70]	.472	5.06 (3,53.98)	**.004**	.202/.579/.488/.346	**<.001/.018/**.626
RMMSD (ms)	6.95 [−5.36, 19.26]	.258	0.60 (3,52.14)	.617	.194/.526/.600/.238	.310/.865/.339
HF (%)	−4.82 [−9,99, 0.34]	.066	0.69 (3,61.82)	.561	.207/.293/**.022**/.154	.822/.181/.344
LF (%)	5.79 [1.28, 10.31]	**.014**	0.48 (3,62.39)	.700	.165/**.016**/**.018**/.076	.301/.973/.546
VLF (%)	−0.94 [−2.88, 1.00]	.327	4.15 (3,68.10)	**.009**	.975/**.004**/.702/.646	**.006**/**.002**/.423
LF/HF	0.98 [0.02, 1.94]	**.046**	0.90 (3,61.42)	.445	.308/.066/**.015**/.355	.435/.566/.151
EDA (μS)	0.16 [−3.03, 3.35]	.917	8.71 (3,43.51)	**<.001**	.538/.230/.490/na	**<.001**/.247/na

SC = stress condition, HR = heart rate, bpm = beats per minute, HF = high-frequency, LF = low-frequency, VLF = very-low-frequency, RMSSD = root mean square of successive differences, EDA = electrodermal activity, B = condition fixed effect unstandardized estimate, CI = confidence interval.

a A = baseline, B = task peak, C = postline, D = pre-sleep.

b Estimated marginal means, Bonferroni-corrected.

### 3.2 Effects of acute stress on sleep

#### 3.2.1 Basic sleep parameters

During the laboratory night, each participant completed a minimum of four sleep cycles (Table 3). Relative to CC, the total proportion of SWS was higher (F(1,30) = 5.685, p = .024, η^2^ = 0.159) and N2 sleep lower (F(1,30) = 13.741, p < .001, η^2^ = 0.314) in SC. This difference between the conditions was driven by the first sleep cycle, where the proportion of SWS was significantly higher (F(1,30) = 9.098, p = .005, η^2^ = 0.233) and proportion of N2 lower (F(1,30) = 20.740, p < .001, η^2^ = 0.409) in SC. The proportion differences of N2 and SWS between the conditions remained significant when the model was adjusted with the duration of the previous night at home. Sleep cycles 2–4 had similar proportions of sleep stages in both conditions (all p > .09, Supplementary Table 1). No sleep differences were found for REMS, SE, WASO, SWS latency and SOL (all p > .14).

**Table 3:** Multivariate ANOVA between the conditions for the basic sleep parameters.

Parameter	SC		CC			
	Mean	SD	Mean	SD	p	p^a^
Laboratory night						
No. of sleep cycles	4.50	0.69	4.71	0.61	.463	.380
TST (min)	387.55	29.36	397.82	27.37	.494	.390
Total N1 (%)	3.06	3.12	2.09	0.75	.449	.290
Total N2 (%)	45.01	4.99	53.02	5.68	**<.001**	**.003**
Total SWS (%)	28.10	4.86	23.54	6.14	**.024**	**.040**
Total REMS (%)	23.83	5.68	21.36	3.98	.364	.542
SE (%)	90.24	6.94	91.88	5.54	.577	.460
SOL (min)	15.05	9.60	19.00	18.99	.399	.673
SWS latency (min)	17.65	8.52	20.07	10.20	.144	.221
WASO (min)	27.03	23.15	16.00	8.36	.142	.163
Cycle 1						
Duration (min)	98.15	18.65	96.32	14.88	.571	.475
N1 (%)	3.75	2.53	3.85	2.48	.846	.940
N2 (%)	32.95	9.52	48.11	11.36	**<.001**	**<.001**
SWS (%)	47.80	11.70	36.81	15.70	**.005**	**.016**
REMS (%)	12.29	6.92	8.10	6.85	.179	.218
WASO (%)	3.23	2.83	3.13	3.30	.586	.992

SC = stress condition, CC = control condition, TST = total sleep time, SWS = slow-wave sleep, REMS = rapid eye movement sleep, SE = sleep efficiency, SOL = sleep onset latency, WASO = wake after sleep onset, SD = standard deviation.

a Adjusted for the duration of previous home night.

#### 3.2.2 EEG power spectral density

**Fig. 3:**
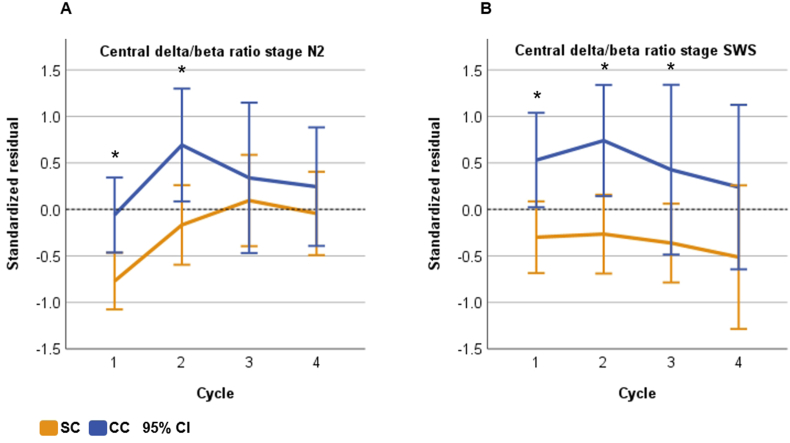
Standardized linear regression residuals of central delta/beta ratio as a function of time (sleep cycles) during N2 (A) and slow-wave sleep (B), adjusted for age and sex. SC = stress condition, CC = control condition, bars refer to 95% confidence interval (CI). **p < .001, *p < .05 for condition pairwise comparison in linear mixed model, Bonferroni-corrected. Beta = (beta-low + beta-high)/2. Delta (0.5–3 Hz), beta-low (16–24 Hz), and beta-high (25–35 Hz).

**Fig. 4:**
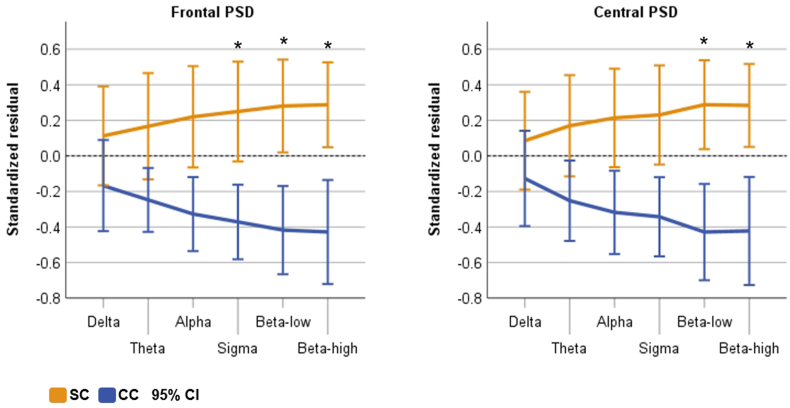
Standardized linear regression residuals of EEG power spectral density (PSD) values averaged over all sleep cycles as function of frequency range, adjusted for age and sex. SC = stress condition, CC = control condition, bars refer to 95% confidence interval (CI). **p < .001, *p < .05 for condition main effect in linear mixed model. Delta (0.5–3 Hz), theta (4–7 Hz), alpha (8–12.5 Hz), sigma (9–16 Hz), beta-low (16–24 Hz), beta-high (25–35 Hz).

**Fig. 5:**
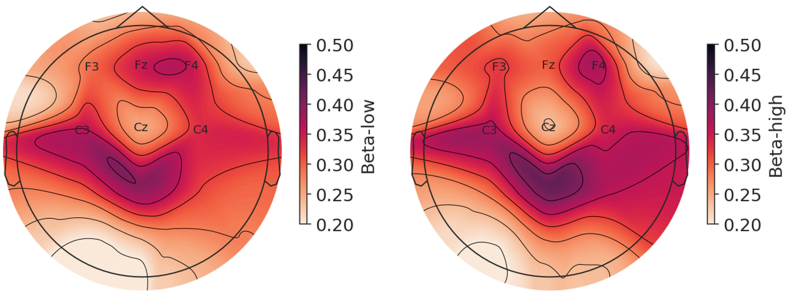
Topographical distribution map of the beta-low and beta-high EEG PSD differences between the conditions in slow-wave sleep. The map displays subtracted values (stress condition minus control condition) calculated with standardized linear regression residuals, adjusted for age and sex. Darker color corresponds to greater difference between the conditions. Reference electrode position was set at FCz. Beta-low (16–24 Hz), beta-high (25–35 Hz).

We found significant condition main effects in both NREMS stages N2 and SWS (Table 4). Delta/beta ratio was significantly reduced during both N2 (frontal F(1,24.116) = 4.308, p = .049; central F(1,25.313) = 3.330, p = .048) and SWS (frontal F(1,27.174) = 8.620, p = .007; central F(1,26.815) = 11.110, p = .003) in SC compared with CC (Fig. 3A–B). During SWS, the condition main effect was significant for the power densities of beta-low (frontal F(1,26.735) = 4.915, p = .035; central F(1,26.783) = 6.486, p = .017) and beta-high (frontal F(1,27.014) = 5.957, p = .021; central F(1,27.075) = 8.218, p = .008) frequencies, such that in SC the PSD was elevated compared with CC (Fig. 4, Fig. 5, Supplementary Fig. 1). In addition, frontal sigma was elevated in SC (F(1,26.620) = 4.230, p = .050) during SWS. Fig. 4 shows a trend where power densities grow with increasing frequency range in SC during SWS, while the reverse is true for CC. In addition, visual inspection implied an earlier onset of the higher frequency activity during SWS in SC (Supplementary Fig. 2). During N2, the condition main effect was also significant for beta-high (frontal F(1,26.907) = 4.967, p = .034; central F(1,26.620) = 5.957, p = .022) and for frontal beta-low (F(1,26.937) = 5.101, p = .032) while close to significance in the central beta-low (F(1,26.714) = 4.183, p = .051). During REMS, we found a significant main effect for frontal theta (F(1,27.000) = 6.996, p = .013) where SC displayed greater activity compared with CC, but other main effects in REMS were non-significant (all p > .07). Regarding the ‘time x condition’ interaction, there was a significant temporal difference during N2 for central delta/beta ratio (F(3,36.826) = 3.473, p = .026, Table 4), where the SC displayed reduced values during sleep cycles 1 and 2 compared with CC (Fig. 3A). Other results for the ‘time x condition’ interactions were non-significant across all sleep stages and frequencies (all p > .07, including cycle 1 vs. 2 contrast), indicating that the overall temporal development of the PSD dynamics were overall similar in both conditions across all sleep stages.

**Table 4:** Linear mixed models between the conditions for the power spectral density over the four sleep cycles.

Position/Frequency	N2			SWS			REMS		
	B [95% CI]	p^a^	p^b^	B [95% CI]	p^a^	p^b^	B [95% CI]	p^a^	p^b^
Frontal									
Delta	.021 [−.017, .059]	.269	.352	.017 [−.014, .048]	.276	.562	.026 [−.002, .053]	.068	.113
Theta	.025 [−.012, .064]	.168	.312	.020 [−.016, .057]	.269	.244	.034 [.008, .061]	**.013**	.321
Alpha	.019 [−.014, .053]	.245	.875	.027 [−.007, .061]	.114	.778	.030 [−.003, .062]	.069	.768
Sigma	.021 [−.009, .051]	.167	.899	.028 [.000, .056]	**.050**	.510	.028 [−.002, .058]	.067	.430
Beta-low	.028 [.003, .054]	**.032**	.338	.027 [.002, .052]	**.035**	.686	.023 [−.006, .051]	.122	.711
Beta-high	.025 [.002, .047]	**.034**	.208	.024 [.004, .045]	**.021**	.472	.021 [−.006, .047]	.119	.945
D/B	−.593 [−1.183, −.004]	**.049**	.125	−.1192 [−2.025, −.359]	**.007**	.610	−.251 [−.747, .245]	.308	.519
Central									
Delta	.017 [−.019, .052]	.348	.352	.012 [−.020, .043]	.462	.319	.024 [−.010, .058]	.158	.304
Theta	.024 [−.011, .059]	.169	.167	.017 [−.017, .050]	.314	.556	.031 [−.001, .064]	.060	.377
Alpha	.016 [−.018, .051]	.335	.750	.024 [−.010, .059]	.163	.914	.012 [−.022, .047]	.468	.296
Sigma	.017 [−.013, .047]	.261	.929	.024 [−.004, .052]	.086	.987	.015 [−.018, .048]	.352	.271
Beta-low	.024 [−.003, .048]	.051	.072	.027 [.005, .050]	**.017**	.965	.016 [−.011, .043]	.242	.486
Beta-high	.025 [.004, .046]	**.022**	.070	.025 [.007, .043]	**.008**	.802	.020 [−.005, .045]	.104	.602
D/B	−.456 [−.907, −.005]	**.048**	**.026**	−1.042 [−1.684, −.400]	**.003**	.717	−.148 [−.528, .232]	.432	.853

B = condition fixed effect unstandardized estimate, CI = confidence interval, SWS = slow-wave sleep, REMS = rapid eye movement sleep, D/B = delta/beta.

Delta (0.5–3 Hz), theta (4–7 Hz), alpha (8–12.5 Hz), sigma (9–16 Hz), beta-low (16–24 Hz), beta-high (25–35 Hz).

a Condition main effect for the stress condition.

b ‘Time x condition’ interaction.

#### 3.2.3 Follow-up analyses with dose-response linear analytics on stress reactivity

**Fig. 6:**
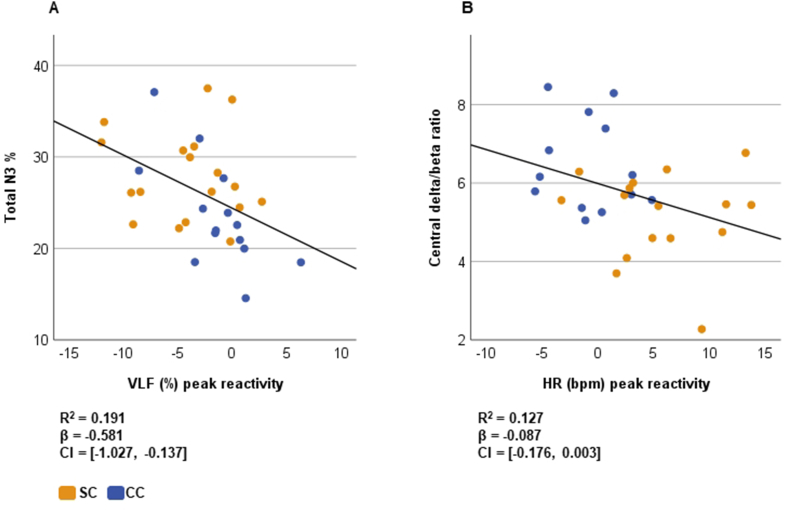
Dose-response linear regression between VLF and proportion of SWS (A) and between heart rate and central delta/beta ratio (B), adjusted for age and sex. β = regression coefficient, CI = confidence interval.

To give a further validation to the results, we conducted analyses with continuous VLF and HR and significant sleep outcomes from the previous analyses, adjusted with sex and age. We found that there was a significant linear relationship between VLF task reactivity and the proportions of total SWS (F(1,30) = 7.100, p = .012, Fig. 6A) and N2 sleep (R^2^ = 0.257, β = 0.777, CI = [0.284, 1.270], F(1,30) = 10.376, p = .003), such that lower VLF predicted increase in SWS and simultaneous decrease in stage N2. In addition, there was a linear relationship close to significance between HR task reactivity and central delta/beta ratio during SWS averaged over the four sleep cycles (F(1,27) = 3.926, p = .058, Fig. 6B), such that an increase in HR predicted dominance of beta activity over delta activity during SWS.

## 4 Discussion

Experimental evidence on the immediate effects of acute stress on sleep is scarce. The current study aimed to fill this gap by experimentally inducing pre-sleep arousal and measuring sleep EEG the following night. First, we observed significant changes in the proportions of sleep stages after acute stress induction such that the duration of SWS was longer in exchange of shorter N2 sleep in SC compared with CC. This effect was taking place predominantly in the first sleep cycle and dissipated in later cycles. Second, we observed elevated PSD in beta-low and beta-high frequency ranges during N2 sleep and SWS. Simultaneous inspection of all frequencies in SWS revealed a pattern where PSD grew towards higher frequencies across all sleep cycles in the stress condition, while the trend was reversed in controls. Inspection of the temporal dynamics of delta/beta ratio in the central region during N2 sleep revealed that the beta activity driven difference in the power ratio between the conditions was greatest during the first half of the night. Additionally, we found significantly elevated frontal theta PSD during REMS in SC.

Our results provide compelling new evidence to elucidate how acute stress influences the sleep of healthy adults. The public speaking stress task in VR used in the current study effectively induced a subjective stress experience as well as a transient physiological stress response. Regarding the subjective experience, participants in both conditions rated the immersivity of the VR similarly, but the stressfulness ratings were significantly higher in SC, suggesting that emotional engagement with the narrative of presenting yourself in front of an audience felt sufficiently authentic in the virtual environment (Dickerson et al., 2004; Gorini et al., 2011). The physiological responses equally validated the stress experience, as we observed elevated heart rate and skin conductance (Critchley, 2002), increased LF power and LF/HF ratio, (Kim et al., 2018) and lower VLF power, (Usui and Nishida, 2017; Shaffer and Ginsberg, 2017) indicating the activation of the sympathetic nervous system in SC. Notably, the physiological response was transient and had dissipated at the pre-sleep period. This may suggest either that cortical arousal instead of physiological arousal underlies the observed effects on sleep, or, that a physiological arousal experienced earlier in the evening carries its effect to sleep independently of the pre-sleep physiological arousal.

Regarding the effects of the stress task on sleep, the observed increase of early SWS in SC was not anticipated when grounding on the evidence from previous stress induction studies in humans. A systematic review from the year 2007 (Kim and Dimsdale, 2007) reported unchanged SWS in the majority of the 27 studies and decreased SWS in a few. The studies that reported decreased SWS used indwelling catheter, hospitalization, and first night laboratory effect as stressors mostly among elderly or sleep apnea patients, (Carvalhaes-Neto et al., 2003; Prinz et al., 2000; Le Bon et al., 2000) and only two studies (n = 10 in both) experimented with a psychoemotional tension protocol involving diminishing and insulting the participant to induce stress (Vein et al., 2002; Levin et al., 2002). Recent overnight (Wuyts et al., 2012; Kim et al., 2020) and napping (Ackermann et al., 2019) studies with stressors comparable to our study also did not report changes in the amount of SWS, but one napping study observed decreased duration of SWS following pre-sleep stress induction (Beck et al., 2022). Interestingly, in line with our findings, two meta-analyses (Stutz et al., 2019; Kredlow et al., 2015) focusing on the effects of pre-sleep physical activity, a potential physiological stress induction, reported the amount of early SWS to increase the subsequent sleep period following physical activity.

However, animal models provide converging evidence to our findings; many studies have presented evidence that social stress promotes SWS, both in terms of increased power and duration (Fujii et al., 2019; Meerlo and Turek, 2001; Wells et al., 2017; Yu et al., 2022). The study on mice by Yu et al. (2022) demonstrated a neural circuit mechanism underlying the increase of restorative sleep after acute stress. They showed how a subpopulation of GABAergic neurons in the ventral tegmental area is transiently activated during social stress induction. This in turn promoted sleep through projections to the lateral hypothalamus and to the paraventricular nucleus, suppressing secretion of corticotropin-releasing factor (CRF), thus promoting SWS sleep. The synaptic homeostasis hypothesis (SHY) (Tononi and Cirelli, 2020) provides further perspectives to a potential homeostatic mechanism. It proposes that net synaptic strength is downscaled during SWS such that its longer duration reflects increased demand for synaptic renormalization, stemming from stress-induced synaptic potentiation during waking (Tononi and Cirelli, 2020; Norimoto et al., 2018). Additionally, evidence suggests that SWS is vital in clearing accumulated waste products from waking-period neural activity, (Xie et al., 2013) and may serve as a sleep pressure mechanism considering that stress further induces activity through excitatory glutamate release (Popoli et al., 2011). Given these points, we suggest that the pressure for SWS in our study may have accelerated through comparable homeostatic mechanisms. This would indicate that the SWS-related adaptations to social stress shown to take place in animal models also occur in humans.

The observed elevation of beta activity in the stress condition was in line with our hypothesis and consistent with several prior experimental studies, including two napping studies with a pre-sleep social stress task, (Ackermann et al., 2019; Beck et al., 2022) a napping study with a learning task, (Schmidt et al., 2006) and an overnight study with non-social hyperarousal induction (Wuyts et al., 2012). Furthermore, elevated beta activity in sleep has also been observed in an experimental study on chronic stress, (Gemignani et al., 2014) and it is a consolidated finding in individuals with insomnia (Riemann et al., 2010; Maes et al., 2014; Zhao et al., 2021). Interestingly, there are also paradoxical observations, such as high-frequency EEG activity during sleep being associated with mindfulness practice, indicating that the nature of persistent cortical arousal through sleep onset may be adaptive or maladaptive depending on the original source of the arousal (Goldstein et al., 2019; Ferrarelli et al., 2013). The heightened cortical arousal in sleep after an acute stress experience may initially function adaptively as an effort to process stress and the related emotional content. However, the restorative function of sleep is disrupted with cortical arousal persisting each night, as exemplified by the hyperarousal model in chronic insomnia (Riemann et al., 2010, 2020). Neurophysiological evidence on the underlying mechanisms of the heightened beta activity during sleep is mixed. The clear majority of studies have focused on individuals with insomnia, and neuroimaging evidence suggests that higher beta activity may arise from impaired GABAergic inhibitory control (Kay and Buysse, 2017). Presence of beta activity in sleep has also been indicated to arise from persistent glucose metabolism in wake-promoting neural circuits (Nofzinger et al., 2004). In addition to our finding that PSD in the beta range was greater in SC over the entire night during SWS, visual inspection of individual sleep cycles revealed a nonsignificant midpoint trend, implying that the natural increase in higher frequency activity towards morning sleep had an earlier onset in SC. That is, the SC showed equal PSD between delta and higher frequencies (ie. alpha, sigma, beta) already in the second sleep cycle, while in CC, this appeared later in the third sleep cycle where, high-frequency activity was already prominent in SC. Although this trend was statistically not significant, it implies that the sleep pressure in SC was alleviated earlier with increased SWS during the first sleep cycle. Regarding the observed elevation of frontal theta PSD during REMS in SC, it may represent heightened hippocampal activity reflecting the processing of emotional memories during sleep as suggested by Hutchison and Rathore (2015) in their review. Interestingly, a study utilizing an acute social stressor proximal to memory encoding found that REMS theta positively interacted with emotional learning outcomes (Kim et al., 2020).

Taken together, we observed two independently occurring sleep consequences following acute stress. As reviewed above, both longer SWS and increased beta activity may have different underlying physiological mechanism. We are then suggesting that sleep operates after an experience of acute stress with two different mechanisms. The first is a homeostatic response serving physiological recovery, observed early in the night. The second, the increase of beta range activity, including the delta/beta ratio, may point to higher cortical arousal during sleep. We suggest that this wake-like interference in sleep may represent the process of emotional adaptation to stress. If the stress exposure is chronic, the persisting cortical arousal in sleep may also become maladaptive, as exemplified by the hyperarousal model of insomnia (Riemann et al., 2010; Bonnet and Arand, 2010).

### 4.1 Strengths and limitations

This study has several strengths. First, the ecological validity of the social stress experience was supported through a state-of-the-art VR solution with a controlled and immersive environment and a credible background narrative, shown to enhance emotional experiences (Diemer et al., 2015; Gorini et al., 2011). VR environments are advantageous in that they offer maximum experimental control and allow for easy and standardized manipulation of contextual factors (e.g. environmental design, behavior of virtual humans). Second, we included multiple physiological parameters to assess level of arousal throughout our study and investigated sleep characteristics with in-depth power spectral density analysis. Finally, this study involved an overnight sleep measurement, allowing temporal effects to be examined in detail. Limitations of this study include the fairly small sample size and the lack of a baseline night to address a potential first night sleep laboratory effect (Agnew et al., 1966). The sleep schedules at home and in the laboratory did not vary across the conditions. However, we did not measure variations in circadian phase and individual chronotype that can influence the sleep measurements. Regarding cortisol measures, our VR task started approximately at 9 p.m., when the circadian cycle of cortisol is at a phase of downregulation, (Weitzman et al., 1971) such that the stress effect in our cortisol measures may have been dampened by this evening descent.

## 5 Conclusion

We suggest that the duration of SWS increased through a homeostatic rebound alleviating the nervous system strain provoked by the earlier stress experience, while elevated beta activity in NREM sleep may represent the stress-induced cortical arousal persisting through sleep onset. Relative to very distressing experiences or chronic states of stress that individuals potentially encounter in their lives, the experimental stress induction in this study was forcedly shorter and milder such that in our study the participants no longer showed physiological activation at bedtime. On the other hand, even the short-term evening stress induction was effective in demonstrating sleep effects, revealing both restorative influence and simultaneous higher cortical arousal. We suggest that both effects potentially function as adaptations to acute stress experiences.

## Funding

This work was supported by the Research Council of Finland (former Academy of Finland, grants 1356020; 1322035; 63056952) and the 10.13039/501100004325Signe and Ane Gyllenberg Foundation.

## CRediT authorship contribution statement

**Emil Hein:** Writing – review & editing, Writing – original draft, Visualization, Methodology, Investigation, Formal analysis, Data curation, Conceptualization. **Risto Halonen:** Writing – review & editing, Validation, Supervision, Methodology, Formal analysis, Conceptualization. **Thomas Wolbers:** Writing – review & editing, Software. **Tommi Makkonen:** Writing – review & editing, Software, Resources. **Markus Kyllönen:** Visualization. **Liisa Kuula:** Writing – review & editing, Supervision, Methodology, Conceptualization. **Ilmari Kurki:** Writing – review & editing, Resources. **Philipp Stepnicka:** Software. **Anu-Katriina Pesonen:** Writing – review & editing, Validation, Supervision, Project administration, Methodology, Funding acquisition, Conceptualization.

## Declaration of competing interest

The authors declare the following financial interests/personal relationships which may be considered as potential competing interests: Thomas Wolbers reports a relationship with neomento GmbH that includes: equity or stocks. Philipp Stepnicka reports a relationship with neomento GmbH that includes: equity or stocks. T.W. and P.S. are stakeholders of neomento GmbH, that develops and sells virtual reality -based psychotherapy applications. They were not involved in data collection, analysis or interpretation of the results at any stage of the study. Their relationship regarding the study is limited to methodology and revision of the final manuscript.
