# Supplementary material for: Does sleep promote adaptation to acute stress: An experimental study
Source: Neurobiol Stress. 2024 Feb 4;29:100613. doi: 10.1016/j.ynstr.2024.100613 (PMC10869260; doi:10.1016/j.ynstr.2024.100613)
Supplement: Multimedia component 1 [file mmc1.docx]

| **Supplementary Table 1**. Multivariate ANOVA between conditions for sleep architecture of sleep cycles 2-4. | | | | | | | | | | |
| --- | --- | --- | --- | --- | --- | --- | --- | --- | --- | --- |
|  | SC | | | CC | | | |  | | |
| Parameter | Mean | SD | | Mean | | SD | | p^1^ | | p^2^ |
| Cycle 2 |  |  | |  | |  | |  | |  |
| Duration (min) | 97.30 | 19.13 | | 88.96 | | 12.42 | | .249 | | .388 |
| N1 (%) | 1.60 | 1.43 | | 1.28 | | 1.28 | | .420 | | .517 |
| N2 (%) | 39.17 | 17.68 | | 42.04 | | 14.94 | | .791 | | .798 |
| N3 (%) | 32.61 | 17.77 | | 35.09 | | 13.83 | | .616 | | .548 |
| REM (%) | 20.17 | 11.46 | | 17.94 | | 10.52 | | .832 | | .578 |
| WASO (%) | 6.45 | 6.99 | | 3.64 | | 2.82 | | .104 | | .193 |
| Cycle 3 |  |  | |  | |  | |  | |  |
| Duration (min) | 101.10 | 20.36 | | 96.79 | | 21.89 | | .534 | | .482 |
| N1 (%) | 4.01 | 8.40 | | 1.71 | | 1.35 | | .447 | | .313 |
| N2 (%) | 43.02 | 12.38 | | 53.56 | | 17.28 | | .089 | | .103 |
| N3 (%) | 16.32 | 9.78 | | 13.00 | | 15.84 | | .797 | | .647 |
| REM (%) | 31.01 | 12.51 | | 27.92 | | 15.74 | | .431 | | .598 |
| WASO (%) | 5.65 | 5.10 | | 3.81 | | 2.55 | | .121 | | .210 |
| Cycle 4 |  | |  | |  | |  | |  | |
| Duration (min) | 86.65 | 16.43 | | 91.00 | | 20.47 | | .465 | | .500 |
| N1 (%) | 2.10 | 3.37 | | 0.81 | | 0.62 | | .456 | | .538 |
| N2 (%) | 51.42 | 14.57 | | 52.12 | | 15.48 | | .876 | | .536 |
| N3 (%) | 13.72 | 13.25 | | 16.67 | | 16.87 | | .798 | | .746 |
| REM (%) | 27.71 | 11.97 | | 27.73 | | 13.78 | | .685 | | .401 |
| WASO (%) | 5.05 | 5.30 | | 2.67 | | 2.17 | | .286 | | .290 |
| SC=stress condition, CC=control condition, TST=total sleep time, SWS=slow-wave sleep, REMS=rapid eye movement sleep, SE=sleep efficiency, SOL=sleep onset latency, WASO=wake after sleep onset, SD=standard deviation.  ^1^Adjusted for sex, age and duration of previous night. | | | | | | | | | | |

| **Supplementary Table 2:** Independent t-test between conditions for the self-reported sleep diary times (15 min accuracy) prior to the lab night. Lab night highlighted, as the values are exact. | | | | | | | | | |
| --- | --- | --- | --- | --- | --- | --- | --- | --- | --- |
|  | Self-reported sleep duration (min±SD) | |  | Bedtime  (hh:mm±SD(min)) | |  | Wakeup time  (hh:mm±SD(min)) | |  |
|  | SC | CC | p | SC | CC | p | SC | CC | p |
| Night pre 4 | 495±75 | 514±78 | .477 | 00:16±52 | 00:46±112 | .371 | 08:31±93 | 09:20±62 | .098 |
| Night pre 3 | 477±83 | 509±45 | .203 | 00:12±57 | 00:23±84 | .637 | 08:09±79 | 08:52±80 | .128 |
| Night pre 2 | 481±72 | 499±45 | .431 | 23:55±47 | 00:24±55 | .106 | 07:56±86 | 08:43±63 | .093 |
| Night pre 1 | 461±39 | 490±50 | .065 | 23:52±48 | 00:25±63 | .091 | 07:33±47 | 08:36±60 | **.002** |
| Mean 1-4 | 479±48 | 503±36 | .117 | 00:03±42 | 00:30±65 | .170 | 08:16±75 | 09:13±42 | **.017** |
| Lab night | 428±8 | 431±9 | .410 | 23:52±11 | 23:54±13 | .619 | 07:01±5 | 07:05±6 | **.030** |
| SD=standard deviation, SC=stress condition, CC=control condition. | | | | | | | | | |


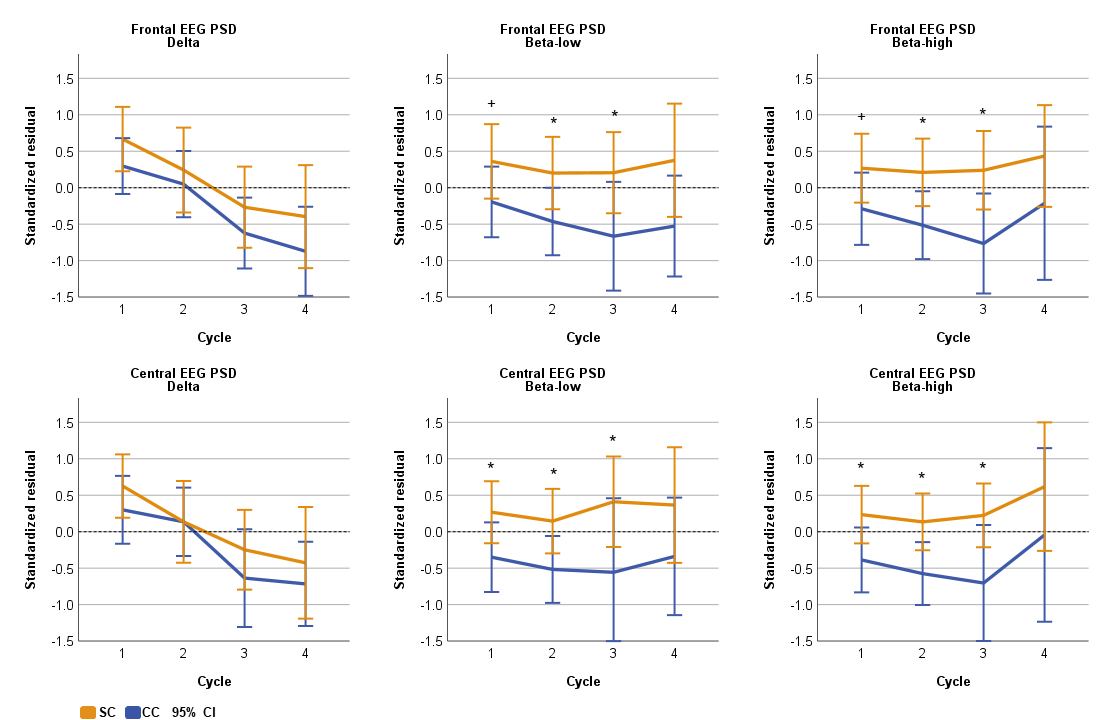


**Supplementary Figure 1:** Standardized linear regression residuals of EEG PSD delta, beta-low, and beta-high frequencies as a function of time (sleep cycle) during slow-wave sleep, adjusted for age and sex. C=stress condition, CC=control condition, bars refer to 95% confidence interval (CI). **p<.001, *p<.05, +p<.07 for condition pairwise comparison in linear mixed model, Bonferroni-corrected.


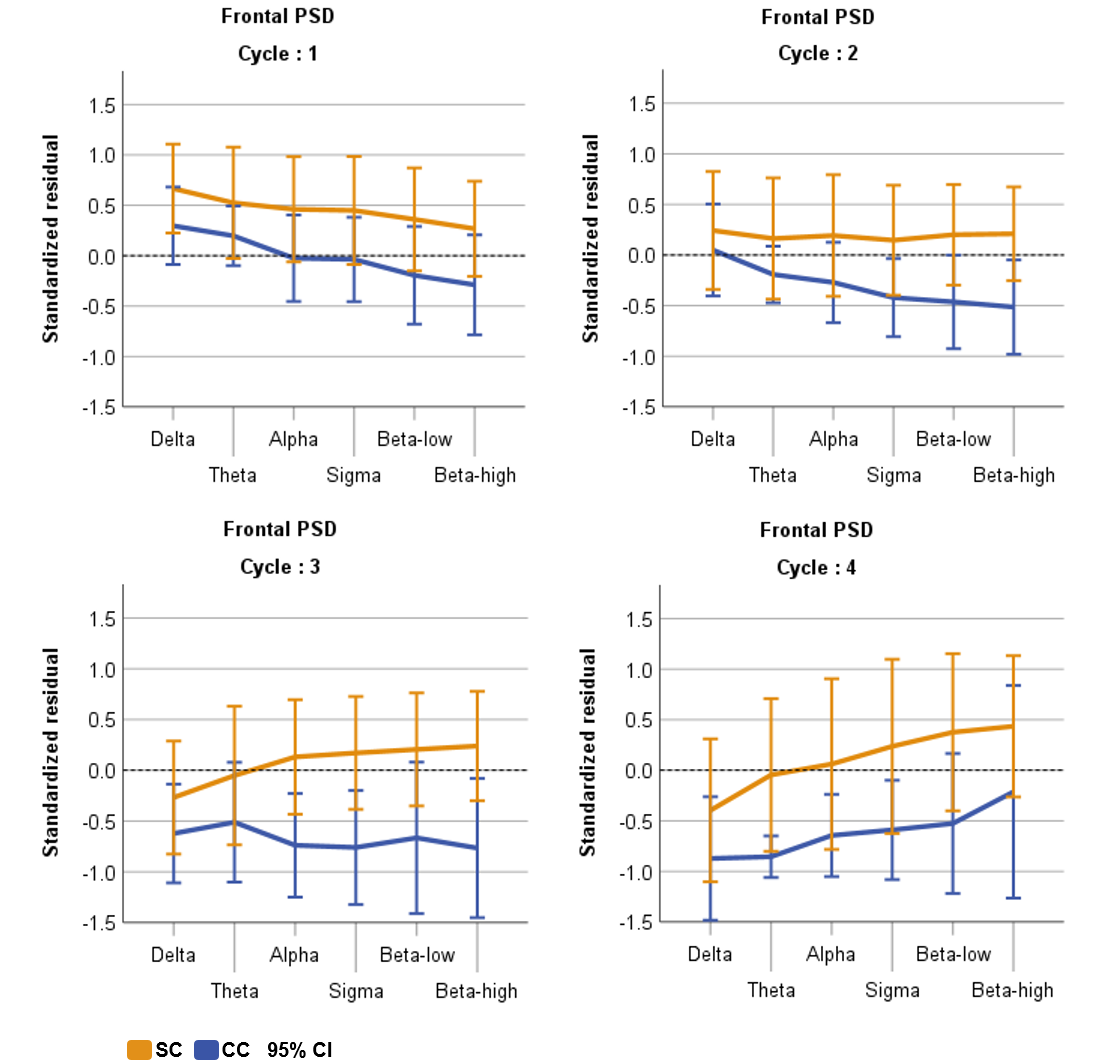


**Supplementary Figure 2:** Standardized linear regression residuals of frontal region slow-wave sleep EEG PSD as a function of frequency range separately for each sleep cycle, adjusted for age and sex. SC=stress condition, CC=control condition, bars refer to 95% confidence interval (CI).
